# Advances and challenges of first-line immunotherapy for non-small cell lung cancer: A review

**DOI:** 10.1097/MD.0000000000036861

**Published:** 2024-01-19

**Authors:** Haiyang Guo, Jun Zhang, Chao Qin, Hang Yan, Xinyue Luo, Haining Zhou

**Affiliations:** a Institute of Surgery, School of Medicine and Life Sciences, Chengdu University of TCM, Chengdu, China; b Suining Central Hospital, An Affiliated Hospital of Chongqing Medical University, Suining, China; c Institute of Surgery, Graduate School, Zunyi Medical University, Zunyi, China.

**Keywords:** biomarkers, chemotherapy, CTLA-4, immune checkpoint inhibitors, NSCLC, PD-1, PD-L1

## Abstract

The current use of immune checkpoint inhibitors (ICIs) for the treatment of lung cancer has dramatically changed the clinical strategy for metastatic non-small cell lung cancer (mNSCLC). As a result of great achievements in clinical trials, 6 programmed death-1 inhibitors (sintilimab, camrelizumab, tislelizumab, pembrolizumab, cemiplimab, and nivolumab), 2 programmed death-ligand 1 inhibitors (sugemalimab and atezolizumab), and 1 cytotoxic T lymphocyte-associated antigen-4 inhibitor (ipilimumab) have been approved as first-line treatment for mNSCLC by the US Food and Drug Administration. Recently, research on ICIs has shifted from a large number of second-line to first-line settings in clinical trials. Results from first-line trials have shown that almost all driver-negative mNSCLC are treated with ICIs and significantly prolong patient survival; however, the low response rate and adverse reactions to immunotherapy remain to be addressed. Here, we summarize the use of ICIs, including monotherapy and combination therapy, in the first-line treatment of mNSCLC in recent years and discuss the low response rate and adverse reactions of ICIs as well as the challenges and expectations for the first-line treatment of mNSCLC in the future.

## 1. Introduction

**Figure 1.**
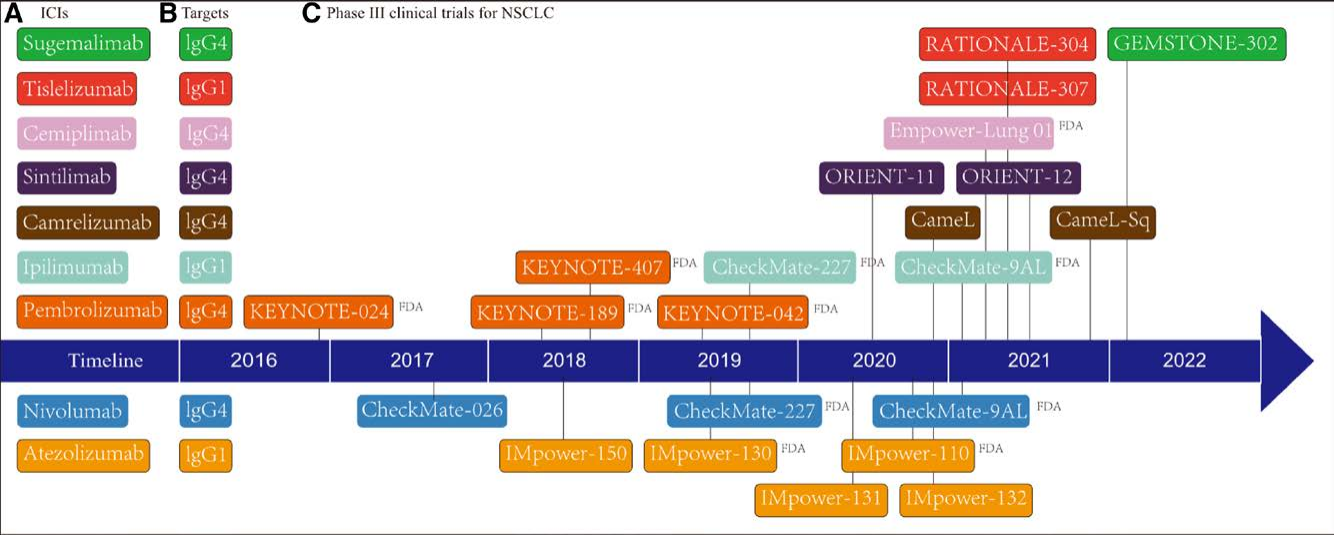
Timeline for clinical trials of first-line immunotherapy for NSCLC; “FDA” represents FDA approval of phase III clinical trials for first-line immunotherapy for NSCLC; (A) ICIs for the first-line treatment of NSCLC; (B) the corresponding targets for ICIs; (C) clinical trials of first-line immunotherapy for NSCLC. FDA = Food and Drug Administration, NSCLC = non-small cell lung cancer.

Lung cancer is the most common cancer globally and the leading cause of cancer-related deaths.^[1]^ The most frequent pathological form of lung cancer is non-small cell lung cancer (NSCLC), which accounts for 85 percent of all lung cancer cases.^[1,2]^ In most cases, lung cancer is treated with radiation, chemotherapy, surgical intervention, and immunotherapy.^[1,2]^ Prior to immunotherapy, platinum-based chemotherapy is the standard treatment for mNSCLC patients.^[3]^ Platinum-based chemotherapy is the cornerstone of treatment for patients with metastatic NSCLC (mNSCLC).^[4]^ Although both standard chemotherapy and immunotherapy can prolong patient survival, immunotherapy in any line of treatment, particularly first-line therapy, promotes survival in patients with mNSCLC.^[5,6]^ mNSCLC has recently been revolutionized by the incorporation of immunotherapy into the standard platinum-based chemotherapy. In NSCLC, immune checkpoint inhibitors (ICIs), a common type of immunotherapy, also known as biologic treatment, have shown clinical success.^[7]^ The 2 main classes of ICIs are programmed death-ligand 1 (PD-(L)1) Inhibitor and cytotoxic T lymphocyte-associated antigen-4 (CTLA-4) inhibitors. T lymphocytes quickly express programmed death-1 (PD-1) on their surfaces after activation, and their ligands are PD-L1 and PD-L2.^[8]^ Binding of T cell surface PD-1 to tumor cell PD-L1 inhibits T cell-associated kinases and prevents cytotoxic T cell responses to tumors. The binding of PD-L1 to its receptor PD-1 on activated T cells reduces antitumor immunity by lowering T cell proliferation, cytokine production, and cytotoxic capacity.^[9]^ In many types of cancer, CTLA-4 is an important negative regulator of T cell responses, eventually resulting in T cell fatigue and T cell malfunction.^[10,11]^ CTLA-4 inhibition can boost antitumor T-cell immunity.^[12]^ ICIs targeting CTLA4, PD-1, and its ligand PD-L1 have shown considerable promise in clinical trials for a range of malignancies, including metastatic melanoma, NSCLC, and renal cell carcinoma (RCC). Currently, immunotherapy, either alone or in conjunction with chemotherapy, is the standard first-line treatment for advanced NSCLC.^[13]^ This review focused on NSCLC. Excluding mNSCLC, which is positive for driver genes, practically all driver-negative mNSCLC patients are now treated with ICIs, which considerably improves patient survival.^[14]^ Here, we discuss the current state of first-line immunotherapy in mNSCLC as well as the challenges and expectations for the first-line treatment of mNSCLC in the future (Fig. 1).

## 2. First-line monotherapy for NSCLC

### 2.1. PD-(L)1 inhibitors

#### 2.1.1. Pembrolizumab.

Pembrolizumab is an anti-PD-1 fully human monoclonal immunoglobulin G4 (IgG4)-kappa isotype antibody that binds to the PD-1 receptor on T cells and prevents PD-1 from interacting with its ligands PD-L1 and PD-L2^[15]^ (Fig. 1). In KEYNOTE-024 [NCT02142738], 305 patients (154 to pembrolizumab, 151 to platinum-based chemotherapy) who had histologically or cytologically confirmed stage IV NSCLC with PD-L1 TPS score of *≥* 50% were included in the group and then they were randomly assigned to pembrolizumab (200 mg Q3W [once every 3 weeks], up to 2 years) or platinum-based chemotherapy (4–6 cycles, Q3W, or crossover to pembrolizumab in the event of disease progression)^[16]^ (Table 1). One distinct improvement in primary end point, median progression-free survival (PFS), was favorable in pembrolizumab group (10.3 months, 95% CI [6.7 to not reached] vs 6.7 months, 95% CI [4.2–6.2] of median PFS; 0.5 [0.37–0.68] of hazard ratio [HR]), the other significant improvement in response rate (RR) (44.8% vs 27.8%) (Table 1). In addition, a lower frequency of grade 3 to 4 treatment-related adverse events (TRAEs, 26.6% vs 53.3%) and a clinically meaningful prolongation of survival were observed. Overall, analysis of this randomized, open, phase III trial showed that pembrolizumab significantly improved PFS and overall survival (OS).^[16]^ Updated analysis of KEYNOTE-024 showed that with prolonged follow-up, first-line pembrolizumab monotherapy continues to demonstrate an OS benefit over chemotherapy in patients with previously untreated advanced NSCLC without epidermal growth factor receptor/anaplastic lymphoma kinase *(EGFR*/*ALK*) aberrations, despite crossover from the control arm to pembrolizumab as subsequent therapy.^[17]^ In a multicenter retrospective analysis, among patients with NSCLC and PD-L1 expression of *≥* 50% treated with first-line pembrolizumab, clinical outcomes were significantly improved in NSCLC with a PD-L1 expression of *≥* 90%.^[18]^ Next to 5 years of follow-up of KEYNOTE-024, pembrolizumab significantly prolonged median OS (26.3 vs 13.4 months; 0.62 [0.48–0.81] of HR) in spite of a crossover rate of 66% in the control arm, and doubled the 5-year OS rate (31.9% vs 16.3%).^[19]^

**Table 1:** Current phase III clinical trials of first-line monotherapy for NSCLC.

Trials	No.	Details of grouping and dosing		Indications		RR(%)	PFS	OS
KEYNOTE-024^[16]^ (1:1)	154	Pembrolizumab (200 mg Q3W for up to 2 yr)	–	Stage IV, no *EGFR*/*ALK*, PD-L1 *≥* 50%		44.8	7.7	26.3
	151	Platinum-doublet chemotherapy (4–6 cycles Q3W)	Pemetrexed in Non-Sq (n = 46)			24.8	5.5	13.4
CheckMate-026^[20]^ (1:1)	211	Nivolumab (3 mg/kg Q2W until PD or toxicity)	–	Stage IV or recurrent NSCLC, PD-L1 *≥* 5%		26.0	4.2	14.1
	212	Platinum-doublet chemotherapy (4–6 cycles Q3W)	Pemetrexed in Non-Sq			33.0	5.9	13.2
KEYNOTE-042^[21]^ (1:1)	637	Pembrolizumab (200 mg Q3W for up to 2 yr)	–	Stage III–IV, No *EGFR*/*ALK*, PD-L1 *≥* 1%	PD-L1 *≥* 1%	39.0	5.5	16.4
							6.8	12.1
	637	Carboplatin plus paclitaxel, or carboplatin plus pemetrexed (Q3W for up to 6 cycles)	Pemetrexed in Non-Sq and at least SD after induction 4 cycles		PD-L1 *≥* 20%	33.0	6.2	18
							6.8	13
					PD-L1 *≥* 50%	27.0	6.5	20
							6.5	12.2
IMpower-110^[22]^ (1:1)	285	Atezolizumab (1200 mg Q3W until PD or toxicity)	–	Stage IV, No *EGFR*/*ALK*, PD-L1 *≥* 1%	PD-L1 *≥* 1%	40.0	5.8	18.9
							5.6	14.7
	287	Sq: platinum plus gemcitabine, or Non-Sq: platinum plus pemetrexed (Q3W for up to 6 cycles)	Sq: BSC Non-Sq: pemetrexed		PD-L1 *≥* 50%	29.0	8.2	20.2
							5.0	14.7
Empower-Lung 1^[23]^ (1:1)	283	Cemiplimab (350 mg Q3W Until PD or 108 wk)	–	Stage IV, No *EGFR*/*ALK*, PD-L1 *≥* 1%		39.0	8.2	/
	280	Sq: platinum plus gemcitabine, or Non-Sq: platinum plus pemetrexed (Q3W for up to 6 cycles)	Sq: BSC Non-Sq: pemetrexed			20.0	5.7	14.2

“/” = not mature or no comparison, BSC = best supportive care, No = number of patients, Non-sq = non-squamous non-small cell lung cancer, NSCLC = non-small cell lung cancer, OS = overall survival, PD = disease progression, PD-L1 = programmed death-ligand 1, PFS = progression-free survival, Q3W = once every 3 wk, RR = response rate, Sq = squamous non-small cell lung cancer.

After KEYNOTE-024, a randomized, open-label, controlled, phase III trial, KEYNOTE-042 [NCT02142738], was launched. The trial included 1274 patients (673 to pembrolizumab, 673 to chemotherapy) with untreated PD-L1 and with a driving mutation in *EGFR*/*ALK* translocation negative, and with the expression of PD-L1 *≥* 1%. They were randomly assigned to receive pembrolizumab (35 cycles, 200 mg Q3W, up to 2 years) or chemotherapy (Q3W, up to 6 cycles) (Table 1). Pembrolizumab group significantly prolonged median OS (16.7 vs 12.1 months; HR, 0.81 [0.71–0.93]). However, the expression in tumors also had an indirect effect on median OS. The best for extended survival was observed in tumors with the expression of PD-L1 TPS *≥* 50%. (20 vs 12.2 months; HR, 0.69 [0.56–0.85]). And The expression of TPS 1% to 49% in pembrolizumab group of median OS was similar to chemotherapy (13.4 vs 12.1; HR, 0.92 [0.77–1.11])^[20]^ (Table 1). With a follow-up of 47 months, first-line pembrolizumab monotherapy significantly improved OS, RR, and PFS outcomes compared with platinum-based chemotherapy in patients with locally advanced/metastatic PD-L1-positive NSCLC without sensitizing *EGFR*/*ALK* alterations, with a manageable safety profile.^[21]^ In light of these findings, the Food and Drug Administration (FDA) has approved pembrolizumab as a first-line treatment for PD-L1 *≥* 1% cancers (Fig. 1).

#### 2.1.2. Atezolizumab.

Atezolizumab is a humanized PD-L1 IgG1 monoclonal antibody (Fig. 1). In the IMpower-110 study (NCT02409342), 572 untreated individuals with confirmed mNSCLC with PD-L1 *≥* 1%, atezolizumab group was found to be superior to platinum-based chemotherapy in terms of survival benefit. In the subset of patients with *EGFR* and *ALK* negative tumors with the highest PD-L1 expression (205 patients), atezolizumab group showed a longer median OS than chemotherapy group (20.2 vs 13.1 months; HR, 0.59 [0.4–0.89]) and an extended median PFS (8.1 vs 5.0 months; HR, 0.63 [0.45–0.88]) and improved RR (38.3% vs 28.6%)^[22]^ (Table 1). Only patients with a high expression of PD-L1 (35%) received non-protocol following ICIs at the time of disease progression in comparison to previous studies, which allowed for a crossover rate. Following a median follow-up of 31.3 months, the latest update exploratory analysis revealed a drop in the median OS benefit, which was most likely due to the control arm better performance (20.2 vs 14.7 months; HR, 0.76 [0.54–1.09]).^[23]^ For patients with high PD-L1-expressing mNSCLC, atezolizumab has also been approved by the FDA for use as a monotherapy (Fig. 1).

#### 2.1.3. Cemiplimab.

The human IgG4 mAb to the PD-1 receptor, cemiplimab, has great affinity and potency (Fig. 1).^[24]^ Recently, in the EMPOWER-Lung 01 [NCT03088540] trial, 563 patients with histologically or cytologically confirmed mNSCLC and their PD-L1 *≥* 50% were randomly assigned to cemiplimab (350 mg Q3W until PD or 108 weeks) or chemotherapy (squamous non-small cell lung cancer: platinum plus gemcitabine, or non-squamous NSCLC: platinum plus pemetrexed [Q3W for up to 6 cycles]). As the condition worsened, a higher percentage of patients might have switched from chemotherapy to cemiplimab. The main outcomes were the OS and PFS rates. Surprisingly, the median PFS was increased 8.2 months and the median OS 14.2 months in the cemiplimab group (HR, 0.54 [0.43–0.68]) with a surprising 74% crossover rate in this experiment. The incidence of 3 to 4 TRAEs of cemiplimab was lower than the cost of chemotherapy (28% vs 39%)^[25]^ (Table 1) retrospective investigation of cancers with high PD-L1 expression (*≥*90%) found a significant survival improvement in the group of tumors that received anti-PD-1 antibodies.^[18]^ In light of the outcomes of the EMPOWER-Lung 01, for mNSCLC patients who have PD-L1 expression *≥* 50%, cemiplimab has been approved by the FDA as first-line treatment (Fig. 1).

#### 2.1.4. Nivolumab.

By binding to the PD-1 receptor, nivolumab inhibits T-cell inhibition caused by cancer cells and their microenvironment, thereby restoring the immune response.^[26]^ In CheckMate-026, 423 patients with untreated stage IV or recurrent NSCLC and a PD-L1 tumor-expression level of 1% or more were randomly assigned in a 1:1 ratio to receive nivolumab (3 mg/kg Q2W until PD or toxicity) or platinum-based chemotherapy (four to 6 cycles Q3W or until pemetrexed in non-squamous NSCLC (n = 46)) (Table 1). The crossover rate from chemotherapy to nivolumab was allowed at the time of disease progression. The primary endpoint was PFS. Nivolumab was not associated with significantly longer PFS than chemotherapy among patients with previously untreated stage IV or recurrent NSCLC with a PD-L1 expression level of 5% or more. OS was similar between the 2 groups. The safety profile of nivolumab was favorable compared to chemotherapy, with no new or unexpected safety signals.^[27]^

### 2.2. CTLA-4 inhibitors

#### 2.2.1. Ipilimumab.

Ipilimumab is a completely humanized IgG1 monoclonal antibody that inhibits CTLA-4^[28]^ (Fig. 1). Currently, there are no clinical trials of ipilimumab as a single agent for the first-line treatment of NSCLC. Ipilimumab plus nivolumab is the first-line therapy for advanced NSCLC.^[29,30]^

#### 2.2.2. Sugemalimab.

The binding of PD-1 to PD-L1 or PD-L2 was prevented by the complete human IgG4 mAb sintilimab, (Fig. 1) which was produced using yeast display technology. Currently, there are no clinical trials of sugemalimab as first-line therapy for NSCLC. In the field of NSCLC, sugemalimumab is usually used in combination with chemotherapy to treat mNSCLC.^[31]^

## 3. First-line monotherapy combinations with chemotherapy for NSCLC

### 3.1. PD-(L)1 inhibitors plus chemotherapy

#### 3.1.1. Pembrolizumab plus chemotherapy.

Recently, the reliable efficacy and safety of pembrolizumab have been demonstrated with the publication of results from a series of phase III clinical trials of pembrolizumab in combination with chemotherapy as first-line treatment for advanced NSCLC. In the randomized study KEYNOTE-407 [NCT02775435],^[32]^ 559 patients with untreated metastatic squamous NSCLC were randomly assigned (1:1) to receive 200 mg of pembrolizumab or saline placebo for up to 35 cycles; all patients also received carboplatin and either paclitaxel or nanoparticle albumin-bound [nab]–paclitaxel for the first 4 cycles. After a median follow-up of 7.8 months, the median OS was 15.9 months (95% CI, [13.2 to not reached]) in the pembrolizumab-combination group and 11.3 months (95% CI, [9.5–14.8]) in the placebo-combination group. The median PFS was 6.4 months (95% CI, [6.2–8.3]) in the pembrolizumab-combination group and 4.8 months (95% CI, [4.3–5.7]) in the placebo-combination group (Table 2). The results showed that treatment with pembrolizumab plus chemotherapy for patients with previously untreated metastatic squamous NSCLC can prolong OS and PFS compared with chemotherapy alone. Adverse events of grade 3 occurred in the pembrolizumab-combination group and in the placebo-combination group were comparable (69.8% vs 68.2%). Subsequently, the investigators reported the health-related quality of life (HRQoL) of patients in KEYNOTE-407.^[32]^ The outcomes of this valuable study demonstrated that pembrolizumab plus chemotherapy maintained or improved HRQoL measurements relative to baseline and improved HRQoL compared to chemotherapy alone at weeks 9 and 18.^[33]^ These results favor the use of pembrolizumab plus chemotherapy as a first-line therapy for metastatic squamous NSCLC. Furthermore, the final analysis of KEYNOTE-407.^[34]^ After positive results from the second interim analysis, patients still receiving placebo could cross over to pembrolizumab monotherapy at the time of confirmed progressive disease. The results of this study were also promising, reaffirming that pembrolizumab plus chemotherapy significantly improves OS and PFS in patients with metastatic squamous NSCLC. In addition, the PFS-2 (time from randomization to progression on next-line treatment/death, whichever occurred first) results support the use of pembrolizumab plus chemotherapy as standard first-line therapy for patients with metastatic squamous NSCLC.

**Table 2:** Current phase III clinical trials of first-line immunotherapy in combination with chemotherapy for squamous NSCLC.

Trials	No.	Details of grouping and dosing		Indications	RR(%)	PFS	OS
KEYNOTE-407^[33]^ (1:1)	278	Pembrolizumab plus carboplatin plus (nab)paclitaxel (4 cycles Q3W)	Pembrolizumab (Q3W up to 31 cycles)	Stage IV, No *EGFR*/*ALK*	59.7	8	17.2
	281	Placebo plus carboplatin plus (nab)paclitaxel (4 cycles Q3W)	Placebo (Q3W up to 31 cycles)		38.4	5.1	11.6
IMpower-131 (1:1:1)^[36]^	338	ACP (4–6 cycles Q3W)	Atezolizumab (Q3W until PD or toxicity)	Stage IV	/	/	/
	343	ACnP (4–6 cycles Q3W)	Atezolizumab (Q3W until PD or toxicity)		49.7	6.3	14.2
	340	CnP (4–6 cycles Q3W)	BSC		41.0	5.6	13.5
CameL-Sq (1:1)^[37]^	193	Camrelizumab plus carboplatin plus paclitaxel (4–6 cycles Q3W)	Camrelizumab (Q3W until PD or toxicity)	Stage IIIB-IV	64.8	8.5	27.4
	196	Placebo plus carboplatin plus paclitaxel (4–6 cycles Q3W)	Placebo (Q3W until PD or toxicity)		36.7	4.9	15.5
RATIONALE-307^[38]^ (1:1)	120	Tislelizumab plus carboplatin plus paclitaxel (4–6 cycles Q3W)	Tislelizumab (Q3W until PD or toxicity)	Stage IIIB/IV	73.0	7.6	/
	119	Tislelizumab plus carboplatin plus (nab)paclitaxel (4–6 cycles Q3W)	Tislelizumab (Q3W until PD or toxicity)		75.0	7.6	/
	121	Carboplatin plus paclitaxel (4–6 cycles Q3W)	BSC		50.0	5.5	/
ORIENT-12^[31]^ (1:1)	357	Sintilimab plus platinum plus gemcitabine (4–6 cycles Q3W)	Sintilimab (Q3W for up to 2 yr)	Stage IIIB/C-IV	45.0	5.5	/
	479	Placebo plus platinum plus gemcitabine (4–6 cycles Q3W)	Placebo (Q3W for up to 2 yr)		35.0	4.9	/
GEMSTONE-302^[32]^ (Sq) (2:1)	129	Sugemalimab plus carboplatin plus paclitaxel (4 cycles Q3W)	Sugemalimab (Q3W up to 35 cycles, PD or toxicity)	Stage IV, No *EGFR*/*ALK*	64.8	8.3	22.8
	63	Placebo plus carboplatin plus paclitaxel (4 cycles Q3W)	Sugemalimab plus pemetrexed (Q3W up to 35 cycles, PD or toxicity)		36.7	4.8	17.7

“/” = not mature or no comparison, ACnP = atezolizumab-carboplatin-nab-paclitaxel, ACP = atezolizumab-carboplatin-paclitaxel, BSC = best supportive care, CnP = carboplatin-nab-paclitaxel, No = number of patients, NSCLC = non-small cell lung cancer, OS = overall survival, PD = Disease progression, PFS = progression-free survival, Q3W = once every 3 wk, RR = response rate, Sq = squamous non-small cell lung cancer.

Additionally, in KEYNOTE-189 [NCT02578680],^[35]^ 616 patients were randomly assigned (2:1) to receive pemetrexed and a platinum-based drug plus either 200 mg of pembrolizumab or placebo every 3 weeks for 4 cycles, followed by pembrolizumab or placebo for up to 35 cycles plus pemetrexed maintenance therapy. Eligible patients were characterized by metastatic non-squamous NSCLC without sensitizing *EGFR* or *ALK* mutations, and had not been previously treated for metastatic disease. The estimated OS at 12 months in the pembrolizumab combination group versus the placebo combination group was 69.2% (95% CI, [64.1–73.8]) vs 49.4% (95% CI, [42.1–56.2]). Median PFS in the pembrolizumab combination group versus the placebo combination group was 8.8 months (95% CI, [7.6–9.2]) vs 4.9 months (95% CI, [4.7–5.5]) (Table 3). Patients in both groups had a comparable probability of grade 3 or higher adverse event. This study showed that pembrolizumab plus chemotherapy prolonged the OS and PFS better than chemotherapy alone in previously untreated patients with metastatic non-squamous NSCLC without *EGFR* or *ALK* mutations. Updated data on September 21, 2018 (median follow-up 23.1 months) showed significantly better updated median OS and median PFS in the pembrolizumab combination therapy arm than in the placebo combination therapy arm.^[36]^ The benefit of pembrolizumab on OS and PFS was confirmed, regardless of PD-L1 expression or the presence of liver/brain metastases. Subsequently, researchers evaluated pre-specified exploratory patient-reported outcomes (PROs) in KEYNOTE-189 patients. The results of this survey showed that the addition of pembrolizumab to standard chemotherapy maintained global health status/quality of life, with improved global health status/quality of life scores at week 21 in the pembrolizumab plus chemotherapy group compared to the placebo plus chemotherapy group.^[37]^

**Table 3:** Current phase III clinical trials of first-line immunotherapy in combination with chemotherapy for non-squamous NSCLC.

Trials	No.	Details of grouping and dosing		Indications	RR(%)	PFS	OS
KEYNOTE-189^[40,42]^ (2:1)	410	Pembrolizumab plus platinum plus pemetrexed (4 cycles Q3W)	Pembrolizumab plus pemetrexed (Q3W up to 31 cycles)	Stage IV, No *EGFR*/*ALK*	47.6	9	22
	206	Placebo plus platinum plus pemetrexed (4 cycles Q3W)	Placebo plus pemetrexed (Q3W up to 31 cycles)		18.9	4.9	10.6
IMpower-150^[45]^ (1:1:1)	402	ACP (4–6 cycles Q3W)	Atezolizumab (Q3W until PD or toxicity)	Stage IV, No *EGFR*/*ALK*	63.5	6.3	19
	400	ABCP (4–6 cycles Q3W)	Atezolizumab plus BVZ (Q3W until PD or toxicity)			8.4	19.5
	400	BCP (4–6 cycles Q3W)	BVZ (Q3W until PD or toxicity)		48.0	6.8	14.7
IMpower-130^[46]^ (2:1)	483	Atezolizumab plus carboplatin plus nab paclitaxel (4–6 cycles Q3W)	Atezolizumab (Q3W until PD or toxicity)	Stage IV, No *EGFR*/*ALK*	49.2	18.6	7
	240	Carboplatin plus nab paclitaxel (4–6 cycles Q3W)	BSC or pemetrexed (Q3W until PD or toxicity)		31.9	13.9	5.5
IMpower-132^[30]^ (1:1)	292	Atezolizumab plus platinum plus pemetrexed (4–6 cycles Q3W)	Atezolizumab plus pemetrexed (Q3W until PD or toxicity)	Stage IV, No *EGFR*/*ALK*	47.0	7.6	17.5
	286	Platinum plus pemetrexed (4–6 cycles Q3W)	Pemetrexed (Q3W until PD or toxicity)		32.0	5.2	13.6
ORIENT-11^[47]^ (2:1)	366	Sintilimab plus platinum plus pemetrexed (4 cycles Q3W)	Sintilimab plus pemetrexed (Q3W for up to 2 yr)	Stage IV, No *EGFR*/*ALK*	51.9	9.2	24.2
	131	Placebo plus platinum plus pemetrexed (4 cycles Q3W)	Placebo plus pemetrexed (Q3W for up to 2 yr)		29.8	5	16.8
RATIONALE-304^[48]^ (2:1)	222	Tislelizumab plus platinum plus pemetrexed (4–6 cycles Q3W)	Tislelizumab plus pemetrexed (Q3W until PD or toxicity)	Stage IIIB/IV, No *EGFR*/*ALK*	57.4	9.7	/
	110	Platinum plus pemetrexed (4–6 cycles Q3W)	Pemetrexed (Q3W until PD or toxicity)		36.9	7.6	/
CameL^[49]^ (1:1)	205	Camrelizumab plus carboplatin plus pemetrexed (4–6 cycles Q3W)	Camrelizumab plus pemetrexed (Q3W until PD or toxicity)	Stage IV, No *EGFR*/*ALK*	61.0	11.3	27.9
	207	Carboplatin plus pemetrexed (4–6 cycles Q3W)	Pemetrexed (Q3W until PD or toxicity)		39.0	8.3	20.5
GEMSTONE-302^[32]^ (Non-sq) (2:1)	191	Sugemalimab plus carboplatin plus pemetrexed (4 cycles Q3W)	Placebo (Q3W up to 35 cycles, PD or toxicity)	Stage IV, No *EGFR*/*ALK*	64.8	9.6	22.8
	96	Placebo plus carboplatin plus pemetrexed (4 cycles Q3W)	Placebo plus pemetrexed (Q3W up to 35 cycles, PD or toxicity)		36.7	5.8	17.7

“/” = not mature or no comparison, ABCP = atezolizumab-bevacizumab carboplatin-paclitaxel, ACP = atezolizumab-carboplatin-paclitaxel, BCP = bevacizumab-carboplatin-paclitaxel, BSC = best supportive care, No = number of patients, Non-sq = non-squamous non-small cell lung cancer, NSCLC = non-small cell lung cancer, OS = overall survival, PD = Disease progression, PFS = progression-free survival, Q3W = once every 3 wk, RR = response rate.

Based on KEYNOTE-189, investigators reported updated efficacy outcomes from protocol-specified final analysis, including outcomes in patients who crossed over to pembrolizumab from pemetrexed-platinum and in patients who completed 35 cycles (up to 2 years) of pembrolizumab.^[38]^ It was concluded that pembrolizumab plus pemetrexed platinum continued to show improved efficacy outcomes with manageable toxicity compared with placebo plus pemetrexed platinum. In addition, a post hoc analysis of KEYNOTE-189 assessed the safety of pemetrexed and platinum in combination with pembrolizumab for the treatment of the same findings.^[39]^

A recent exploratory analysis retrospectively evaluated the outcomes of patients with advanced NSCLC.^[40]^ The investigators pooled data from patients with advanced NSCLC in KEYNOTE-021 cohort G (non-squamous), KEYNOTE-189 (non-squamous), and KEYNOTE-407 (squamous) to evaluate the efficacy of pembrolizumab in combination with platinum-based chemotherapy in patients with NSCLC and stable brain metastases. In patients with brain metastases, the median OS was 18.8 months with pembrolizumab in combination with chemotherapy and 7.6 months with chemotherapy, and the median PFS was 6.9 and 4.1 months, respectively. Pembrolizumab plus chemotherapy had a higher objective response rate (ORR) and longer duration of response than chemotherapy alone, regardless of the brain metastasis status. The outcomes of all the above phase III clinical trials and subsequent updated data analysis support pembrolizumab plus chemotherapy as a first-line treatment for advanced/metastatic NSCLC.

#### 3.1.2. Tislelizumab plus chemotherapy.

Tislelizumab is an investigational humanized IgG4 mAb that binds to the extracellular domain of human PD-1 with high specificity and affinity and blocks the binding of both PD-L1 and PD-L2^[41]^ (Fig. 1).

With the publication of the outcomes of phase III clinical trials associated with tislelizumab, tislelizumab plus chemotherapy has shown great potential as a first-line treatment for advanced NSCLC. RATIONALE-304 [NCT03663205]^[42]^ was an open-label phase III trial. Eligible patients were randomized to arm A (n = 222) and arm B (n = 110), who had histologically confirmed stage IIIB to IV non-squamous NSCLC. Arm A received tislelizumab plus platinum (carboplatin or cisplatin) and pemetrexed every 3 weeks, whereas arm B received platinum and pemetrexed alone Q3W during induction treatment, followed by intravenous maintenance pemetrexed Q3W. With a median study follow-up of 9.8 months, not only was the PFS for tislelizumab plus chemotherapy significantly longer compared with chemotherapy alone (9.7 vs 7.6 months, HR, 0.645 [0.462–0.902], *P* = .0044), but the RR was higher and the duration of response was longer (Table 3). The outcomes of this study support tislelizumab plus chemotherapy as a first-line treatment option for nonsquamous NSCLC. RATIONALE-307 [NCT03594747],^[43]^ a phase III clinical trial, aimed to assess the efficacy, safety, and tolerability of tislelizumab plus chemotherapy versus chemotherapy alone as first-line treatment for patients with histologically confirmed stage IIB/IV squamous NSCLC. Patients were randomly assigned (1:1:1) to receive one of the following regimens: tislelizumab plus paclitaxel plus carboplatin (arm A), tislelizumab plus nab-paclitaxel plus carboplatin (arm B), or paclitaxel and carboplatin (arm C). After a median study follow-up of 8.6 months (95% CI, [8.1–9.0]), the PFS of both arm A and B was 7.6 months, while the PFS of arm C was 5.5 months ((HR, 0.524 [0.370–0.742], P 0.001 [A vs C])) and (0.478 [0.336–0.679], *P* < .001 [B vs C]) (Table 2). Finally, this study demonstrated that the addition of tislelizumab to chemotherapy as a first-line treatment for patients with advanced squamous NSCLC significantly prolonged PFS and had a good safety/tolerability profile, regardless of PD-L1 expression.

#### 3.1.3. Camrelizumab plus chemotherapy.

Camrelizumab (SHR-1210, anti-PD-1 antibody) is a high-affinity, humanized, IgG4-kappa PD-1 mAb that has significant anti-tumor effects, including in lung cancers^[44]^ (Fig. 1). A recent double-blind, randomized phase III trial called CameL-sq [NCT03668496]^[45]^ investigated the efficacy and safety of camrelizumab or placebo plus chemotherapy as first-line treatment for patients with stage IIIB–IV squamous NSCLC. Eligible patients were randomized (1:1) to receive 4 to 6 cycles of carboplatin plus paclitaxel with camrelizumab or placebo (every 3 weeks), followed by maintenance therapy with camrelizumab or placebo. The results showed that camrelizumab plus carboplatin plus paclitaxel significantly prolonged PFS (median, 8.5 vs 4.9 months, *P* < .0001) and OS (median, not reached vs 14.5 months, *P* < .0001) compared with placebo chemotherapy (Table 2). These results support the use of camrelizumab plus chemotherapy as a first-line treatment for advanced squamous NSCLC. Another randomized, open-label, multicenter, phase 3 trial called CameL [NCT03134872]^[46]^ was used to evaluate the efficacy of camrelizumab plus chemotherapy in non-squamous NSCLC in China. Eligible patients had non-squamous NSCLC without *EGFR* and *ALK* alterations, and had no previous systemic chemotherapy. Patients were randomly assigned (1:1) to receive carboplatin plus pemetrexed plus camrelizumab (n = 205) or carboplatin plus pemetrexed (n = 207) every 3 weeks for 4 to 6 cycles, respectively, followed by maintenance treatment with either camrelizumab plus pemetrexed or pemetrexed alone. The median follow-up duration was 11.9 months (IQR 9.0–14.9). PFS in this interim analysis was significantly prolonged with camrelizumab plus chemotherapy compared to chemotherapy alone (median, 11.3 months, 95% CI [9.6–15.4] vs 8.3 months [6.0–9.7], HR 0.60 [0.45–0.79], one-sided *P* = .0001) (Table 3). The outcomes of this study support the use of camrelizumab plus carboplatin and pemetrexed as first-line treatment options for Chinese patients with advanced non-squamous NSCLC without *EGFR* and *ALK* alterations.

#### 3.1.4. Atezolizumab plus chemotherapy.

Atezolizumab is a humanized IgG1 mAb against PD-L1, which showed clinical benefits when combined with chemotherapy as a first-line treatment for NSCLC (Fig. 1). Impower-150 [NCT02366143],^[47]^ an open-label, phase III study, evaluated atezolizumab plus chemotherapy for metastatic non-squamous NSCLC in patients who had not previously received chemotherapy. Eligible patients were randomly assigned to receive atezolizumab plus carboplatin plus paclitaxel (ACP), bevacizumab plus carboplatin plus paclitaxel (BCP), or atezolizumab plus BCP (ABCP) every 3 weeks for 4 or 6 cycles, followed by maintenance therapy with atezolizumab, bevacizumab, or both. The outcomes showed that the median PFS was longer in the ABCP group than in the BCP group (8.3 vs 6.8 months, HR for PD or death, 0.62 [0.52–0.74] *P* < .001) (Table 3). In the final Impower-150 OS analysis,^[48]^ ACP showed numerically, but not statistically significant, OS improvement compared to BCP. Updated data with an additional 20 months of follow-up showed continued OS improvement with ABCP versus BCP in all patients. In addition, researchers confirmed the reliable tolerability and controllability of ABCP in first-line non-squamous NSCLC compared with ACP and BCP by analyzing the safety and PROs of Impower-150.^[49]^ In Impower-130 [NCT02367781],^[50]^ eligible patients were randomly assigned (2:1) to receive atezolizumab plus chemotherapy (carboplatin plus nab-paclitaxel) or chemotherapy alone for 4 or 6 21-day cycles followed by maintenance therapy. The outcomes showed significant improvements in the median OS (18 months; 95% CI [16.0–21.2]) in the atezolizumab plus chemotherapy group and 13. Months [12.0–18.7] in the chemotherapy group; stratified HR = 0.79 (95% CI [0.64, 0.98], *P* = .33) and median PFS (7. 0 months, 95% CI [6.2–7.3]) in the atezolizumab plus chemotherapy group and 5.5 months [4.4–5.9] in the chemotherapy group; stratified HR 0.64 (95% CI [0.4–0.7], *P* < .001) (Table 3). Impower-131 [NCT02367794],^[51]^ PFS improvement with atezolizumab plus carboplatin plus nab-paclitaxel (A + CnP) (n = 343) versus carboplatin plus nab-paclitaxel (CnP) (n = 340) was seen in the intention-to-treat (ITT) population (median, 6.3 vs 5.6 months; HR 0.71 [0.60–0.85] *P* = .0001). OS improvement with atezolizumab plus carboplatin plus nab-paclitaxel (A + CnP) (n = 343) versus carboplatin plus nab-paclitaxel (CnP) (n = 340) was observed in the PD-L1high subgroup (HR 0.48 [0.29–0.81]), despite not being formally tested. The addition of atezolizumab to platinum-based chemotherapy significantly improved the PFS of patients with first-line squamous NSCLC (Table 2). Researchers have reported the final outcomes of the phase III Impower-132 study [NCT02657434],^[29]^ which assessed atezolizumab plus carboplatin or cisplatin plus pemetrexed (APP) in patients with non-squamous NSCLC. At the primary PFS analysis (May 22, 2018; median follow-up, 14.8 months), APP exhibited significant PFS improvement versus PP (median 7.6 vs 5.2 months, stratified HR 0.60 [0.49–0.72], *P* < .0001). The final analyses (July 18, 2019, median 17.5 vs 13.6 months, stratified HR = 0.86 [0.71–1.06], p 0.1546) (Table 3). OS and PFS results favored APP versus PP across subgroups.

#### 3.1.5. Sintilimab plus chemotherapy.

Sintilimab, an anti-PD-1 mAb,^[52]^ showed significant antitumor effects by blocking the interaction between PD-1 and its ligand (Fig. 1). Recently, a phase 1b study [NCT02937116]^[53]^ showed the good efficacy and manageable toxicity of sintilimab plus chemotherapy in patients with non-squamous and squamous NSCLC. ORIENT-11 [NCT03607539],^[54]^ a randomized, double-blind, phase III study, was designed to compare the efficacy and safety of sintilimab with those of placebo, both in combination with chemotherapy. Patients were randomized (2:1) to receive either sintilimab 200 mg or placebo plus pemetrexed and platinum once every 3 weeks for 4 cycles, followed by sintilimab or placebo plus pemetrexed therapy. Crossover or treatment beyond disease progression was also allowed. As of November 15, 2019, the median follow-up was 8.9 months. The median PFS was significantly longer in the sintilimab-combination group than that in the placebo-combination group (8.9 vs 5.0 months, HR 0.482 [0.362–0.643] *P* < .00001) (Table 3). The confirmed ORR was 51.9% (95% CI [45.7%–58.0%]) in the sintilimab-combination group and 29.8% (95% CI [22.1%–38.4%]) in placebo-combination group. The incidence of grade 3 or higher adverse events was 61.7% in sintilimab-combination group and 58.8% in placebo-combination group. This PFS showed that sintilimab plus chemotherapy was superior to chemotherapy alone in Chinese patients with previously untreated, locally advanced, or metastatic nonsquamous NSCLC. Therefore, sintilimab plus chemotherapy has potential as a first-line treatment for nonsquamous NSCLC. Subsequently, the updated OS data of ORIENT-11 demonstrated that in non-squamous NSCLC, the addition of sintilimab to chemotherapy significantly prolonged the OS.^[55]^ ORIENT-12 [NCT03629925],^[30]^ a randomized, double-blind, phase III study, was used to compare the efficacy and safety of sintilimab with placebo combined with gemcitabine (GP). Eligible patients with locally advanced or metastatic squamous NSCLC without *EGFR*-sensitive mutations or *ALK* rearrangements were randomized (1:1) to receive 200 mg sintilimab or placebo plus GP every 3 weeks for 4 or 6 cycles, followed by sintilimab or placebo as maintenance therapy until disease progression or 2 years (Table 2). Between September 25, 2018, and July 26, 2019, random assignment to the sintilimab-GP group (n = 179) and placebo-GP group (n = 178) was performed. After a median follow-up period of 12.9 months, sintilimab-GP continued to show a meaningful improvement in PFS compared to placebo-GP (HR 0.536 [0.422–0.681], *P* < .00001). There was no significant difference in the incidence of grade 3 or worse treatment-induced adverse events or the incidence of treatment-induced adverse events leading to death among patients in the 2 treatment groups. Therefore, based on the PFS of this study, sintilimab plus GP reveals more clinical benefit than GP alone as first-line therapy in patients with locally advanced or metastatic squamous NSCLC. Thus, the toxicity was acceptable.

#### 3.1.6. Sugemalimab plus chemotherapy.

Sugemalimab is an IgG4 mAb against PD-L1^[56]^ and has demonstrated significant antitumor effects in NSCLC in recent phase III clinical trials (Fig. 1). GEMSTONE-301 [NCT03728556],^[30]^ a randomized, double-blind, phase III clinical trial, aimed to evaluate the efficacy and safety of sugemalimab in patients with stage III NSCLC whose disease had not progressed after concurrent or sequential chemoradiotherapy. Patients were randomly assigned (2:1) to receive sugemalimab 1200 mg or a matching placebo administered intravenously every 3 weeks for up to 24 months. At data cutoff (March 8, 2021), median follow-up was 14.3 months (IQR 6.4–19.4) for patients in the sugemalimab group and 13.7 months (7.1–18.4) for patients in the placebo group. PFS was significantly longer with sugemalimab than with placebo (median 9.0 months, 95% CI [8.1–14.1] vs 5.8 months, 95% CI [4.2–6.6], stratified HR 0.64 [0.48–0.85] *P* = .0026). The short-term outcomes of this study were satisfactory and set the stage for the treatment of NSCLC with sugemalimab plus chemoradiotherapy. In GEMSTONE-302 [NCT03789604],^[31]^ eligible patients had histologically or cytologically confirmed stage IV squamous or non-squamous NSCLC without known *EGFR* sensitizing mutations, *ALK*, ROS1, or RET fusions, no previous systemic treatment for metastatic disease. Patients were randomly assigned (2:1) to 2 main groups, namely the sugemalimab group and placebo group (Table 2). The former received sugemalimab plus platinum-based chemotherapy (carboplatin and paclitaxel for squamous NSCLC or carboplatin and pemetrexed for non-squamous NSCLC). The latter group received placebo plus the same platinum-based chemotherapy regimens for squamous or non-squamous NSCLC as the sugemalimab group. This study had a median follow-up of 17.8 months (IQR 15.1–20.9) in the final analysis (March 15, 2021), with PFS sugemalimab group versus placebo group of (median 9.0 months, 95% CI [7.4–10.8] vs 4.9 months [4.8–5.1]; stratified HR 0.48 [0.39–0.60] *P* < .0001). Not surprisingly, regardless of PD-L1 expression, PFS was significantly prolonged and maintained with sugemalimab plus chemotherapy compared with placebo plus chemotherapy, suggesting an emerging strategy for the first-line treatment of squamous and non-squamous metastatic NSCLC is coming soon.

## 4. First-line dual immunotherapy for NSCLC

### 4.1. PD-(L)1 inhibitors plus CTLA-4 inhibitors

#### 4.1.1. Nivolumab plus ipilimumab.

CheckMate-227 (NCT02477826), a phase III study, was divided into 2 parts based on the expression of PD-L1. One part of the study (Part 1a) enrolled patients with PD-L1 expression ≥ 1% and the other part (Part 1b) enrolled patients who had the expression of PD-L1 < 1%, respectively. In part 1a, 1189 patients with the expression of PD-L1 ≥ 1% were randomly assigned (in 1:1:1 ratio) to nivolumab (3 mg/kg Q2W) plus ipilimumab group (1 mg/kg Q6W up to 2 years) (n = 396), nivolumab monotherapy group (240 mg Q2W) (n = 396), or platinum-doublet chemotherapy group (Q3W up to 4 cycles) (n = 397). In Part 1b, 550 patients with PD-L1 expression < 1% were randomly assigned (in 1:1:1 ratio) to nivolumab (360 mg Q3W) plus ipilimumab (1 mg/kg Q6W up to 2 years) group, nivolumab in combination with platinum-doublet chemotherapy (Q3W up to 4 cycles) group, or only platinum-doublet chemotherapy (Q3W up to 4 cycles) (Table 4).^[57]^ Patients with PD-L1 expression received nivolumab in combination with ipilimumab, whereas those without the expression of PD-L1 received only chemotherapy alone or chemotherapy in combination with nivolumab. Regardless of PD-L1 expression status, nivolumab in combination with ipilimumab significantly improved OS when compared to chemotherapy (HR 0.76 [0.65–0.90] for PD-L1 expression positive, HR 0.64 [0.51–0.81] for PD-L1 expression negative). Although the combination has already been approved by the FDA for patients with PD-L1 expression-positive NSCLC, the survival benefits of nivolumab in combination with ipilimumab in people with PD-L1 expression negative are clinically significant.^[57]^

**Table 4:** Current phase III clinical trial of first-line dual immunotherapy for NSCLC.

Trials	No.	Details of grouping and dosing		Indications	RR(%)	PFS	OS
CheckMate-227^[58]^ (1:1:1)	396	Nivolumab 3 mg/kg Q2W plus ipilimumab 1 mg/kg Q6W for up to 2 yr	–	Stage IV, No *EGFR*/*ALK*, PD-L1 *≥* 1%	36.0	5.6	17.1
	396	Nivolumab monotherapy, chemotherapy according to histologic subtype, 4 cycles Q3W	Pemetrexed maintenance Q3W for Non-Sq until PD		30.0	5.1	14.9
	397	Nivolumab 3 mg/kg Q2W for up to 2 yr	–		/	/	/
	187	Nivolumab 3 mg/kg Q2W plus ipilimumab 1 mg/kg Q6W for up to 2 yr	–	Stage IV, No *EGFR*/*ALK*, PD-L1 < 1%	27.0	5.6	17.2
	177	Nivolumab plus chemotherapy, chemotherapy according to histologic subtype, 4 cycles Q3W	Pemetrexed maintenance Q3W for Non-Sq until PD		21.0	4.7	12.2
	186	Nivolumab 3 mg/kg Q2W plus chemotherapy according to histologic subtype, 4 cycles Q3W	Nivolumab ± pemetrexed maintenance Q3W for Non-Sq until PD		/	/	/
CheckMate-9AL^[59]^ (1:1)	361	Nivolumab 360 mg Q3W plus ipilimumab 1 mg/kg Q6W plus 2 cycles Q3W chemotherapy	Nivolumab 360 mg Q3W plus ipilimumab 1 mg/kg Q6W for up to 2 yr (±pemetrexed)	Stage IV, No *EGFR*/*ALK*	38.0	6.7	15.8
	358	Platinum-based chemotherapy according to histologic subtype (4 cycles Q3W)	Pemetrexed (Q3W in Non-Sq until PD)		25.0	5.3	11.8

“/” = not mature or no comparison, No = number of patients, Non-sq = non-squamous non-small cell lung cancer, NSCLC = non-small cell lung cancer, OS = overall survival, PD = Disease progression, PD-L1 = programmed death-ligand 1, PFS = progression-free survival, Q2W = once every 2 wk, Q3W = once every 3 wk, Q6W = once every 6 wk, RR = response rate.

After a median of 54.8 months of follow-up, nivolumab in conjunction with ipilimumab had a better OS than chemotherapy in patients with the expression of PD-L1 ≥ 1% (HR 0.76 [0.65–0.90]). The 4-year OS rate was 29% versus 18% for nivolumab in combination with ipilimumab against chemotherapy for PD-L1 *≥* 1% and 24% versus 10% for PD-L1 < 1%. 4-year OS rate with nivolumab plus ipilimumab versus chemotherapy was 29% versus 18% (PD-L1 ≥ 1%) and 24% versus 10% (PD-L1 < 1%). Both squamous and non-squamous histology benefited from treatment. In such a descriptive study, the efficacy of nivolumab with ipilimumab was found to be superior to that of nivolumab alone (PD-L1 *≥* 1%) and nivolumab combined with chemotherapy (PD-L1 < 1%). The safety of the study was similar to that previously reported.^[58]^ Nivolumab in combination with ipilimumab was authorized by the FDA in 2020 as a first-line treatment for patients with metastatic NSCLC who had a PD-L1 *≥* 1% (Fig. 1). This approval was based on the findings of CheckMate-227 for NSCLC.^[59]^

#### 4.1.2. Negative results for dual immunotherapy.

The primary end points of the phase III MYSTIC [NCT02453282] study, which was an improvement in OS with durvalumab compared to chemotherapy and an improvement in OS or PFS with durvalumab plus tremelimumab compared to chemotherapy, were not observed in patients whose tumors expressed PD-L1 *≥* 25% of their cells. Based on exploratory analysis, a bTMB threshold of 20 mutations per megabase was found to be necessary for optimum OS improvement with durvalumab in combination with tremelimumab.^[60]^ PROs showed that durvalumab reduced symptoms and improved the time to deterioration of PROs in patients with mNSCLC, but had no negative effects on quality of life. Although MYSTIC failed to meet its primary endpoint in mNSCLC patients with PD-L1 expression *≥* 25%.^[61]^

For mNSCLC patients with PD-L1 *≥* 50%, the KEYNOTE-598 [NCT03302234] examined the combination of pembrolizumab and ipilimumab and found no benefit in either OS or PFS (PFS HR 1.06, *P* = .72; OS HR 1.08, *P* = .74).^[62]^ In other words, adding ipilimumab to pembrolizumab as a first-line treatment for mNSCLC with PD-L1 *≥* 50% and without EGFR/ALK aberrations does not increase efficacy. As a result, the safety monitoring committee decided to call off the study and the participants ceased receiving ipilimumab and placebo medication.

## 5. First-line dual immunotherapy combinations with chemotherapy for NSCLC

### 5.1. PD-(L)1 inhibitors plus CTLA-4 inhibitors plus chemotherapy

#### 5.1.1. Nivolumab plus ipilimumab plus chemotherapy.

CheckMate-9LA [NCT03215706] included 719 individuals, regardless of PD-L1 expression, with histologically proven stage IV or recurrent disease who were randomly assigned to receive either nivolumab plus ipilimumab in combination with chemotherapy (2 cycles Q3W, n = 361) or platinum-based chemotherapy (4 cycles Q3W, n = 358) based on histological subtype. The major endpoint OS of the trials was met (median 15.8 vs 11.0 months; HR, 0.72 [0.61–0.86]) (Table 4). According to the findings of this Phase III clinical study, the combination treatment of nivolumab plus ipilimumab with chemotherapy for NSCLC was approved by the FDA^[63]^ (Fig. 1). The most recent updated follow-up data from the study, which was conducted for a period of 2 years, further validates the prolonged survival benefit of dual immunotherapy combinations with chemotherapy for NSCLC, and 2-year OS and PFS benefits persisted regardless of PD-L1 expression and histological types.^[64]^

POSEIDON [NCT03164616], a comparable study, randomly allocated 1013 patients with mNSCLC to 1 of 3 arms: durvalumab plus tremelimumab in combination with chemotherapy, durvalumab in combination with chemotherapy, or standard chemotherapy alone. According to a recent press release, the addition of durvalumab and tremelimumab to chemotherapy resulted in an improvement in OS and PFS compared with chemotherapy alone. In the near future, there will be an analysis of primary data, which will provide a crucial opportunity to directly compare chemotherapy plus PD-L1 inhibitors and quantify the benefit of adding CTLA-4 inhibitors.

## 6. Toxicity

Generally, there is no perfect treatment. ICIs exert antitumor effects by specifically blocking immune checkpoint molecules and their ligands. In fact, the main function of immune checkpoint molecules is to balance the effects of the immune system, which is extremely important for maintaining normal immune function. So, it is not difficult to understand that immune ICIs can cause immune-related adverse events (irAEs) to the body while fighting against tumors. Therefore, despite the many advantages of immunotherapy in terms of efficacy, the adverse events it causes to patients cannot be ignored. According to reported clinical trials, irAEs associated with anti-PD-1 and anti-PD-L1 include rash, pruritus, diarrhea, decreased appetite, nausea, enteritis, pneumonia, and inflammatory arthritis.^[65,66]^

Currently, the incidence of adverse events is lower with monotherapy than standard chemotherapy. For example, the outcomes of updated analysis of KEYNOTE-024 showed a lower incidence of grade 3 to 5 adverse events associated with pembrolizumab treatment than with chemotherapy (31.2% vs 53.3%).^[17]^ In KEYNOTE-042,^[20]^ the comparison of treatment-related adverse events of grade 3 or worse in the pembrolizumab group (n = 636) and chemotherapy group (n = 252) was 18% versus 41%, and the number of deaths was 13 and 14, respectively (2% vs 2%). In CheckMate-026,^[27]^ grade 3 or 4 treatment-related adverse events occurred in 18% and 51% of the nivolumab group and chemotherapy groups, respectively. However, treatment-related adverse events associated with immunotherapy plus chemotherapy are usually higher than those associated with chemotherapy alone. For example, the results from analysis of KEYNOTE-021, KEYNOTE-189, and KEYNOTE-407 showed that incidence of adverse events associated with pembrolizumab plus chemotherapy versus chemotherapy was 88.2% versus 82.8% in patients with brain metastases, and 94.5% versus 90.6% in those without.^[40]^ This outcome may be due to the superimposed effects of drug toxicity.

As toxic reactions are inevitable, timely interventions for toxic reactions are important. Recommendations of the American Society of Clinical Oncology clinical practice guideline are as follows^[66]^: For most grade 2 toxicities, treatment with ICIs can be suspended and treated with corticosteroids, and ICIs can be resumed when symptoms revert to grade 1 or less. Grade 3 toxicities usually require suspension of ICIs and initiation of high-dose corticosteroids. In general, permanent discontinuation of ICIs is recommended for grade 4 toxicities, with the exception of endocrinopathies that have been controlled by hormone replacement. Therefore, although multiple phase III clinical trials have shown that ICIs significantly prolong PFS and OS with manageable toxicity, further exploration is required to reduce irAEs and manage toxic drug reactions.

In addition, a few studies have found that ICIs may enhance the acceleration of tumor growth, referred to as hyperprogressive disease (HPD). In advanced NSCLC, the incidence of HPD ranges from 13% to 25.7%.^[67]^ However, the definition of HPD remains controversial, and there is a paucity of research and reports on immunotherapy for HPD. Despite the numerous advantages of immunotherapy, its toxic effects cannot be ignored.

## 7. How to select patients based on PD-L1 expression

The addition of immunosuppressants to standard first-line chemotherapy has led to a dramatic change in the treatment of advanced or mNSCLC, with both single and combination immunotherapy significantly prolonging patient PFS. How can the best way of immunotherapy be selected to benefit patients in clinical practice?

### 7.1. PD-L1 ≥ 50%

Patients with PD-L1 50% have a variety of treatment options. The pembrolizumab group showed a significant improvement in median OS compared to the chemotherapy group (30 vs 14.2 months) in KEYNOTE-024.^[16]^ The OS of patients with PD-L1 levels > 50% in the pembrolizumab group was higher than that in the chemotherapy group (20 vs 12 months) in the KEYNOTE-042.^[20]^ Pembrolizumab had a HR for OS benefit of 0.9 in the KEYNOTE-024 subgroup of nonsmokers as compared to chemotherapy for never-smokers and in the KEYNOTE-042 was 1.1.^[16,20]^ It is possible that this indicates that there is no survival benefit associated with the use of pembrolizumab in never-smokers as compared to chemotherapy. Therefore, considering both Keynote-189 and Keynote-407, we recommend combining pembrolizumab with chemotherapy for the treatment of never smokers with NSCLC.^[32,35]^ This could be a more effective approach for dealing with them. In addition, based on the significant median OS observed in IMPOWER-110, the FDA gave approval for the marketing of atezolizumab as a first-line treatment for mNSCLC patients with PD-L1 *≥* 50%.^[22]^

### 7.2. PD-L1 1% to 49%

Attractively, Pembrolizumab was indicated for the treatment of NSCLC based on the findings of KEYNOTE-042, in particularly for smokers’ males with PD-L1 levels ranging from 1% to 49%.^[20]^ Moreover, pembrolizumab in combination with chemotherapy can be used to treat patients without smoking, critically ill individuals, and those with liver metastases.^[35,36]^ According to IMpower-130, atezolizumab in combination with standard chemotherapy can also be used as a treatment option for NSCLC with PD-L1 1% to 49%.^[50]^ According to CheckMate 9LA, adding chemotherapy to nivolumab in combination with ipilimumab can also be used as a treatment option for NSCLC in patients under the age of 75.^[68]^

### 7.3. PD-L1 < 1%

According to CheckMate-227, combination nivolumab with ipilimumab can only be used to treat patients with PD-L1 < 1%.^[57]^ Pembrolizumab and chemotherapy could be an excellent option for many patients with PD-L1 < 1%. According to KEYNOTE-407 and updated KEYNOTE-189, patients with PD-L1 level of <1% had the choice of being treated with either atezolizumab or pembrolizumab in combination with chemotherapy.^[32,36]^

## 8. Drug resistance mechanism

In addition to toxicity, resistance to ICIs is another significant challenge facing current immunotherapy for NSCLC. Tumor immunotherapy resistance is the result of interactions between the host, tumor cells, and the tumor microenvironment (TME).

### 8.1. Tumor microenvironment

The TME consists of blood vessels, immune cells, fibroblasts, bone marrow-derived inflammatory cells, various signaling substances, and an extracellular matrix. Previously considered bystanders of carcinogenic effects, these host cell and extracellular matrix components are now believed to play important roles in tumorigenesis, therapeutic resistance, and other processes.^[69]^

#### 8.1.1. Positive regulation of TME.

In the early stages of the tumor, the recruitment and/or activation of immune cells and associated matrix components by tumor cells in the TME creates a specific anti-tumor inflammatory microenvironment that slows tumor development.^[70]^ With the development of the tumor and gradual downregulation of the immune function of the body, the function of the TME is exhausted after the continuous stimulation of tumor antigens and immune activation. Inadequate effector cell infiltration, dysfunction, depletion, and impaired memory cell formation may result in their inability to perform normal functions or even transform into a cancer-promoting phenotype, resulting in the negative regulation of the TME.^[70,71]^

#### 8.1.2. Negative regulation of TME.

In the progression of the tumor, especially in the advanced stage, tumor-infiltrating immune cells in the TME, such as Treg cells, myeloid-derived suppressor cells, M2 macrophages, and other suppressors in the TME, contribute to immunotherapy resistance.^[72,73]^ These cells can restrict effector T-cell function and promote recruitment and initiation of immunosuppressive cells by secreting cytokines, including interleukin-10, tumor necrosis factor α, and interferon gamma.^[74]^

Moreover, the TME contains non-immune cells associated with carcinogenesis, tumor recurrence, and metastasis, which are associated with treatment resistance. Among them, cancer-associated fibroblasts not only secrete CXCL12 and TGF-β to block the attraction and stimulation of T cells^[75]^ but also release IL-6, IL-1, VEGF, and CCL2 to inhibit anti-tumor immunity and promote the formation of Tregs,^[76]^ leading to immune resistance.

### 8.2. Tumor cell

#### 8.2.1. Targetable oncogenic drivers.

Most oncogenic drivers of NSCLC, including EGFR-activating mutations, ALK rearrangements, ROS1 gene fusions, Kirsten rat arcomaviral oncogene homolog, RET rearrangement, and MET amplification, have all been linked to monoresistance.^[77]^

Clinical trials have shown that NSCLC with EGFR mutations does not respond to immune monotherapy, possibly because when the EGFR signaling pathway is activated, inhibitory cytokines are produced that allow Tregs to multiply, which weakens the immune system function.^[78]^ NSCLC with ALK fusion can decrease neoantigen formation through the PI3K-AKT and MEK-ERK pathways and increase the number of immunosuppressive cells, leading to low efficacy of immunotherapies.^[79]^ In addition, Kirsten rat arcomaviral oncogene homolog mutations may induce immune escape through upregulation of neoantigen expression.^[80]^

#### 8.2.2. Tumor mutation burden.

Tumor mutation burden is the relative number of mutations in a given tumor tissue.^[81]^ If there are more non-synonymous mutations in the tumor, more neoantigens appear, and the PD-1/PD-L1 axis is involved in blocking the immune response, thus affecting the response of tumor cells to ICIs.

In recent years, several phase III randomized controlled trials have similarly shown the predictive role of tumor mutation burden in efficacy.^[82–84]^ In a study of NSCLC treated with pembrolizumab, it was found that these patients developed treatment resistance.^[85]^ This may be due to the loss of 7 to 18 mutation-associated neoantigens that produce effective responses and complex de novo mutations.^[85]^

#### 8.2.3. Major histocompatibility complex variation.

The major histocompatibility complex is a highly dynamic set of genes encoding antigen presentation and T-cell activators that are important in the immune response and control.^[86]^

McGranahan et al found that HLA mutations are a pervasive immune escape mechanism in NSCLC progression in 2017.^[87]^ The inactivation of B2M genes leads to reduced or missing expression of major histocompatibility complex Class I molecules, making them unrecognized by CD8 T cells the main cause of immunotherapy resistance in NSCLC.^[88]^

#### 8.2.4. Epigenetics.

In recent years, a deeper understanding of epigenetic research has led to a focus on the involvement of various mechanisms, such as DNA methylation, non-coding RNA expression, and post-transcriptional regulation in NSCLC.^[89]^ These mechanisms play crucial roles in the development of NSCLC. Epigenetic mutations in NSCLC are expected to be effective markers for the diagnosis, staging, and prognosis of NSCLC. On the other hand, epigenetic changes can also promote invasive behaviors in NSCLC that can become resistant to immunotherapy.^[90]^

### 8.3. Host

In addition to factors in the tumor and its microenvironment, the patient age, BMI, smoking history, presence of other underlying conditions such as high blood pressure and diabetes, and use of other physical or/and chemotherapy may affect the effectiveness of immunotherapy.^[91]^

## 9. Discussion

### 9.1. Challenges and prospects in the future

Although first-line randomized phase III clinical trials have shown significant effectiveness and the FDA has approved some ICIs for the treatment of NSCLC, (Fig. 1) there is still more clinical research on new targets or combinations to improve immunotherapy (Table 5).

**Table 5:** Advances in clinical trials of ICIs in the future.

Targets	Trials	NCT.	Phase	No.	ICIs and it combinations
PD-(L)1	CHOICE-01	NCT03856411	III	465	Toripalimab
	JAVELIN Lung 100	NCT02576574	III	1224	Avelumab ± chemotherapy
	CANOPY-1	NCT03631199	III	673	Pembrolizumab + chemotherapy ± canakinumab
	KEYLYNK-006	NCT03976323	III	1005	Pembrolizumab + chemotherapy + olaparib
	KEYLYNK-008	NCT03976362	III	857	Pembrolizumab + carboplatin + taxane ± olaparib
	ZEAL-1L	NCT04475939	III	650	Pembrolizumab ± niraparib
	LEAP-006	NCT03829319	III	726	Chemotherapy + pembrolizumab ± lenvatinib
	LEAP-007	NCT03829332	III	623	Pembrolizumab ± lenvatinib
	LEAP-008	NCT03976375	III	405	Lenvatinib ± pembrolizumab
	INSIGNA	NCT03793179	III	846	Pembrolizumab + carboplatin ± pemetrexed
TIGIT	SKYSCRAPER-01	NCT04294810	III	635	Atezolizumab ± tiragolumab
	KEYVIBE-003	NCT04738487	III	1246	Pembrolizumab ± vibostolimab
	SKYSCRAPER-06	NCT04619797	II	500	Chemotherapy + atezolizumab ± tiragolumab
LAG3	/	NCT04623775	II	520	Chemotherapy + nivolumab ± relatlimab

ICIs = immune checkpoint inhibitors, LAG3 = Lymphocyte-activation gene 3, NCT = NCT Number, No = Number Enrolled, PD-(L)1 = inhibitors and new combinations, PD-(L)1 = programmed death-(ligand) 1, TIGIT = T-cell immunoreceptor with Ig and ITIM domains.

#### 9.1.1. Programmed death-ligand 1.

Toripalimab, a humanized anti-PD-1 monoclonal antibody, has been approved for the treatment of previously treated advanced metastatic melanoma and nasopharyngeal carcinoma that has already been treated. However, CHOICE-01, a phase III trial in China to study toripalimab, shifted to NSCLC. According to CHOICE-01,^[92]^ toripalimab in combination with first-line chemotherapy increased PFS.^[93]^ According to these findings, toripalimab plus chemotherapy is a better first-line treatment option than chemotherapy alone for patients with NSCLC who do not have driver genetic abnormalities.^[93]^

Avelumab is a fully human anti-PD-L1 IgG1 antibody that has shown promising efficacy and an acceptable safety profile in multiple tumor types, including NSCLC.^[94,95]^ The JAVELIN Lung 100 trial [NCT02576574], a phase III, open-label, multicenter trial of avelumab (an anti-PD-L1 antibody) compared with platinum-based doublet chemotherapy as first-line treatment for recurrent or stage IV NSCLC with PD-L1 expression positive and without *EGFR* mutation and *ALK* translocation.^[96]^ Although the JAVELIN Lung 200 trial reported that the avelumab group did not show improved OS in PD-L1-positive patients with platinum treatment compared to the chemotherapy group, the trial had a favorable safety profile.^[97]^ Will avelumab be a new option for NSCLC without *EGFR/ALK* mutations? The results of the first trial were expected.

CANOPY-1, a novel combination, mainly evaluates pembrolizumab in combination with platinum-based doublet chemotherapy, with or without canakinumab, in previously untreated locally advanced or metastatic NSCLC. The trial primarily included patients with stage IIIB to IV NSCLC. In 2022, AACR reported that canakinumab matching-placebo in combination with pembrolizumab and platinum-based doublet chemotherapy did not improve PFS or OS in the first-line treatment of patients with advanced NSCLC.

Several trials have evaluated Poly ADP-Ribose Polymerase inhibitors plus chemotherapy and PD-1 inhibitor (a new combination) for first-line NSCLC (KEYLYNK-006 [NCT03976323], KEYLYNK-008 [NCT03976362], and ZEAL-1L [NCT04475939]). Both KEYLYNK-006 (pembrolizumab with maintenance olaparib or maintenance pemetrexed) and KEYLYNK-008 (pembrolizumab with or without maintenance olaparib) were used as first-line treatments for metastatic squamous NSCLC. ZEAL-1L is a multicenter, randomized, double-blind, placebo-controlled study of niraparib plus pembrolizumab versus placebo plus pembrolizumab as maintenance therapy in patients with advanced or mNSCLC.

The LEAP program is a novel combination of 13 tumor types, including lung cancer. This combination has yielded positive results in advanced RCC and endometrial cancer, and has been approved by the FDA for appropriate indications.^[98,99]^ Three studies involving lung cancer were conducted in the LEAP program: LEAP-006 [NCT03829319], LEAP-007 [NCT04676412], and LEAP-008 [NCT03976375]. LEAP-006 is a phase III study exploring pembrolizumab plus lenvatinib versus pembrolizumab plus chemotherapy plus placebo for the treatment of first-line squamous NSCLC. This trial was divided into 2 phases, with the primary endpoint in phase 1 being safety and the primary endpoints in phase 2 being PFS and OS.^[100]^ The results of run-in in LEAP-006 reported the safety of this combination (n = 13) with 69.2% ORR, and first imaging assessment achieved satisfactory results, and the tumors was not growth in this combination of patients.^[101]^ LEAP-007 is a phase III study exploring pembrolizumab plus lenvatinib versus pembrolizumab plus placebo for the first-line treatment of NSCLC with TPS *≥* 1%. The primary endpoint of this study was PFS and the PFS of Pembrolizumab plus lenvatinib was 6.6 months compared with pembrolizumab plus placebo (PFS 6.6 vs 4.2 months). ORR has nearly doubled (from 20.7% to 40.5%), which is fully comparable to KEYNOTE-189 (47.6%),^[35]^ which suggesting that the combination is largely a strong tumor shrinker and offers a new idea for the first-line treatment of NSCLC with TPS *≥* 1%, with a nearly 3 times reduction in disease progression rate (from 27.7% to 10.4%), suggesting that lenvatinib offers an idea for addressing primary resistance to immunotherapy alone. Unfortunately, even though this combination benefited ORR and PFS, there was no advantage in OS of 14.1 months (vs 16.4 months). It is speculated that these results may be related to toxicity caused by the drug dose. LEAP-008 is a phase III study exploring pembrolizumab plus lenvatinib versus docetaxel plus lenvatinib for the treatment of NSCLC with disease progression after platinum-containing or immunotherapy plus chemotherapy. It should be noted that the LEAP program did not involve studies of squamous carcinoma, perhaps in view of safety or awaiting the results of LEAP-008.

A phase III trial study (EA5163/S1709 INSIGNA) [NCT03793179] explored pembrolizumab as a first-line treatment to follow pemetrexed and car-boplatin ± pembrolizumab after disease progression, and then compared it with pembrolizumab, pemetrexed, and carboplatin to follow pembrolizumab and pemetrexed maintenance for the treatment of patients with stage IV non-squamous NSCLC. This trial is recruiting and we look forward to its final findings.

#### 9.1.2. T-cell immunoreceptor with Ig and ITIM domains.

Currently, multiple trials are actively evaluating another target: a T-cell immunoreceptor with Ig and ITIM domains. Tiragolumab is an ICIs used against T-cell immunoreceptor with Ig and ITIM domains. The multiple trials included 2 phase III studies (SKYSCRAPER-01 [NCT04294810] and KEYVIBE-003 [NCT04738487]) and 1 phase II study of SKYSCRAPER-06 [NCT04619797].

SKYSCRAPER-01 is aim to evaluate how well tiragolumab plus atezolizumab compared with Placebo plus atezolizumab in previously untreated locally advanced unresectable or metastatic PD-L1-selected NSCLC. KEYVIBE-003 is assessing the primary endpoint OS of pembrolizumab ± vibostolimab and SKYSCRAPER-06 is exploring efficacy, safety, and pharmacokinetics of chemotherapy + atezolizumab ± tiragolumab.

#### 9.1.3. Lymphocyte activation gene 3.

Lymphocyte activation gene 3 (LAG3) is a potential first-line treatment for NSCLC. Recently, the FDA officially approved an anti-LAG3 mAb (relatlimab) in combination with nivolumab for the treatment of metastatic melanoma, which is the world first approved LAG3 antibody drug. Recently, a phase II study [NCT04623775], PD-1 plus chemotherapy with or without LAG3 (n = 520), reported positive results, which may expand recent positive results in first-line melanoma plus PD-1 inhibitors.

#### 9.1.4. Others.

Imprime PGG has shown promising clinical efficacy in combination with mAb treatment for cancer.^[102]^ LUN15-017 [NCT03003468] is an open-label, multi-institutional, single-arm phase II cohort study of pbrolizumab and imprime PGG. APX005M is a CD40 agonist antibody.^[103]^ Recently, 2 early-stage studies ([NCT03123783] and [NCT03502330]) related to APX005M have been conducted for the treatment of NSCLC and other cancers. One study investigated APX005M in combination with nivolumab in patients with NSCLC or metastatic melanoma, and the other studied APX005M in combination with nivolumab and cabiralizumab for the treatment of patients with advanced melanoma, NSCLC, or RCC. We look forward to the publication of more results and subsequent studies on APX005M focusing on its efficacy and safety. Recently, a study [NCT05117242] was initiated to investigate the safety and efficacy of GEN1046 as a single agent or in combination with ICIs for the treatment of recurrent (non-small cell) lung cancer. Tu, McGlinchey, Wang, Martin, Ching, Floc’h, Kurasawa, Starrett, Lazdun, Wetzel, Nuttall, Ng, Coffman, Smith, Politi, Cooper and Streicher^[104]^ think anti-PD-L1 combination with anti-CD73 promotes T cell response for *EGFR*-mutated NSCLC. This makes Anti-PD-L1 plus anti-CD73 therapy possible for the treatment of *EGFR*-mutated NSCLC.

In summary, a number of clinical trials are ongoing involving avelumab, toripalimab, tiragolumab or relatlimab in combination with or without chemotherapy and some studies aiming to explore safety and efficiency of PD-(L)1 in combination with lenvatinib, Poly ADP-Ribose Polymerase, APX005M, and GEN1046 for advanced or metastatic NSCLC. The results of these trials are looking forward to success. In the future, new targets, combinations, and treatment sequences must continue to be explored for the first-line treatment of locally advanced unresectable or metastatic NSCLC.

## Author contributions

**Conceptualization:** Haiyang Guo, Jun Zhang, Chao Qin.

**Formal analysis:** Haiyang Guo.

**Investigation:** Jun Zhang.

**Methodology:** Chao Qin.

**Project administration:** Haining Zhou.

**Resources:** Hang Yan.

**Visualization:** Hang Yan.

**Writing – original draft:** Haiyang Guo, Jun Zhang, Haining Zhou.

**Writing – review & editing:** Haiyang Guo, Xinyue Luo, Haining Zhou.
